# Confirmation of the Tibetan Brown Bear (*Ursus arctos pruinosus*) in Central Nepal Based on Genetic and Camera‐Trap Evidence

**DOI:** 10.1002/ece3.74400

**Published:** 2026-10-07

**Authors:** Rishi Baral, Ram Bahadur Gurung, Abhisek Sapkota, Naresh Subedi, Anthony J. Giordano, Rajan Prasad Paudel, Rabin Kadariya, Gopal Khanal, Kinoshita Kodzue, Toshio Tsubota, Michito Shimozuru

**Affiliations:** ^1^ Laboratory of Wildlife Biology and Medicine, Department of Environmental Veterinary Science, Graduate School of Veterinary Medicine Hokkaido University Sapporo Japan; ^2^ Himalayan Nepal Program The Society for the Preservation of Endangered Carnivores and their International Ecological Study (SPECIES) Kathmandu Nepal; ^3^ One Step Co., Ltd., Program and Research Section Nagoya Aichi Japan; ^4^ National Trust for Nature Conservation Lalitpur Nepal; ^5^ Institute of Forestry Tribhuvan University Pokhara Nepal; ^6^ S.P.E.C.I.E.S.‐The Society for the Preservation of Endangered Carnivores and their International Ecological Study Ventura California USA; ^7^ Center for Human‐Carnivore Coexistence Colorado State University Fort Collins Colorado USA; ^8^ Department of National Parks and Wildlife Conservation (DNPWC) Government of Nepal Kathmandu Nepal; ^9^ Graduate School of Asian and African Area Studies Kyoto University Kyoto Japan; ^10^ One Health Research Center Hokkaido University Sapporo Japan

**Keywords:** Brown bear, camera‐trapping, Nepal, non‐invasive genetics, trans‐Himalayas, *Ursus arctos pruinosus*

## Abstract

The brown bear ( *Ursus arctos* ) persists in small, fragmented populations across the Himalayan region; however, its subspecific identity and distribution in Nepal have long remained uncertain due to the absence of genetic evidence. Here, we integrated non‐invasive genetic analyses with systematic camera‐trap surveys to resolve the taxonomic status of brown bears in Nepal's trans‐Himalayan landscapes, and document their physical occurrence there. We collected fecal and hair samples from Dolpa District and the Upper Mustang region between 2022 and 2024, and conducted camera‐trapping in both Upper Mustang (Annapurna Conservation Area) and Dolpa Districts as part of broader mammal and snow leopard surveys. We used microsatellite genotyping to identify unique individuals and avoid duplicate sampling; however, subspecies‐level assignment relied primarily on the use of Mitochondrial DNA (i.e., a control‐region). We determined subspecific identity based on sequence similarity and phylogenetic clustering with reference haplotypes of Tibetan ( *Ursus arctos pruinosus* ) and Himalayan (*U. a. isabellinus*) brown bears. We found that our phylogenetic analyses consistently placed all samples from Nepal inside the Tibetan brown bear lineage, forming a well‐supported clade with reference sequences from the Tibetan Plateau, and providing the first genetic confirmation of *U. a. pruinosus* in Nepal. Our camera traps recorded five independent capture events at three different stations between 3834 and 4816 m *a.s.l*., and documented bear activity three different habitats: alpine steppe, shrubland, and grasslands. A verified photographic record we obtained from the Manaslu Conservation Area further supports the occurrence of Tibetan brown bears beyond their primary genetic sampling sites. These findings establish the first molecular baseline for brown bears occurring in Nepal, and highlight the conservation significance of its trans‐Himalayan landscapes as peripheral habitats for a globally rare, and nationally Critically Endangered, brown bear subspecies.

## Graphical abstract

Using non‐invasive genetic analyses and systematic camera‐trap surveys, we provide the first genetic confirmation of the Tibetan brown bear ( *Ursus arctos pruinosus* ) in Nepal. Phylogenetic analyses of mitochondrial DNA placed all Nepalese samples within the Tibetan Plateau lineage, distinct from the Himalayan brown bear (*U. a. isabellinus*). Camera‐trap detections across trans‐Himalayan habitats further demonstrate the occurrence and conservation importance of this globally rare and nationally Critically Endangered subspecies in central Nepal.

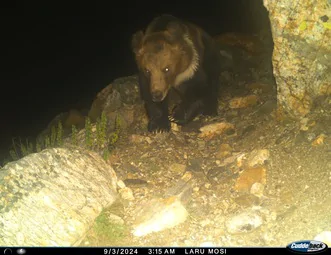

## 1 Introduction

The brown bear ( *Ursus arctos* ) is one of the most widely distributed terrestrial mammalian carnivores. It occupies a broad range of habitats across North America, Europe, and Asia, from temperate forests to alpine and high‐altitude ecosystems (Hunter 2020; McLellan et al. 2017). In Asia, brown bear range extends into the Himalayas and the Tibetan Plateau, where it persists at elevations exceeding 5000 m, often under extreme climatic and ecological conditions meters (McLellan et al. 2017; Nawaz et al. 2014). Despite this broad geographic distribution, populations in the Himalayan region are typically small, fragmented, and poorly documented, largely due to the logistical challenges of conducting surveys, the remoteness of study areas, and limited capacity for long‐term monitoring (Swenson et al. 2011).

Although globally the brown bear is listed as Least Concern by the IUCN (McLellan et al. 2017), this rangewide status masks pronounced regional declines and localized threats. Across much of their Asian range, brown bears face increasing pressures from habitat fragmentation, human–wildlife conflict, infrastructure development, and illegal killing (Swenson et al. 2021). In Nepal, the species is classified as Critically Endangered; with fewer than 20 mature individuals estimated nationwide, it has a highly restricted and discontinuous distribution confined to the northern high Himalayas (Jnawali et al. 2011). The species receives full legal protection under the National Parks and Wildlife Conservation Act (1973), yet effective conservation is hindered by limited ecological and taxonomic information (Government of Nepal 1973).

Two subspecies of brown bear are generally recognized as occurring in the Himalayan–Tibetan region: the Himalayan brown bear (*U. a. isabellinus*), largely restricted to the northwestern Himalaya of Pakistan and northern India, and the Tibetan brown bear (*U. a. pruinosus*), believed to be distributed across the Tibetan Plateau and adjoining high‐altitude regions of China and Nepal (Galbreath et al. 2007; Lan et al. 2017; Pocock 1941; Servheen 1999). Mitochondrial DNA analyses have demonstrated that the Tibetan and Himalayan brown bears represent genetically distinct evolutionary lineages, despite their geographic proximity, supporting their recognition as separate subspecies (Galbreath et al. 2007; Lan et al. 2017). These subspecies are considered geographically and genetically distinct, yet both are rare, sparsely distributed, and insufficiently studied (McLellan et al. 2017; Swenson et al. 2021). In Nepal, many historical records of brown bears have relied on indirect sign, local reports, or photographic evidence alone, frequently without genetic confirmation; this has resulted in long‐standing uncertainty regarding the identity of subspecies (Chetri 2008, 2022; Kusi et al. 2024).

Recent camera‐trap surveys have improved documentation of brown bear occurrence in northwestern Nepal and confirmed the species' presence at extreme elevations and in arid alpine landscapes, including in districts such as Humla (Kusi et al. 2021). However, evidence from central Nepal, including the Upper Mustang region of the Annapurna Conservation Area (ACA), has remained limited and largely unverified at the genetic level (Aryal et al. 2010; Aryal, Hopkins, et al. 2012; Chetri 2008). This lack of confirmation has impeded efforts to clarify the distributional limits of *U. a. pruinosus* and to develop region‐specific conservation strategies.

Genetic approaches, particularly mitochondrial DNA (mtDNA) analysis, provide a powerful tool for resolving taxonomic ambiguity and identifying subspecific lineages in elusive and low‐population‐density carnivores (Galbreath et al. 2007; Lan et al. 2017). When combined with systematic camera‐trapping surveys, genetic data can not only confirm which subspecies is present but also strengthen inferences about habitat use, community context, and predator‐prey interactions in remote mountain systems. Integration of these methods is especially valuable in landscapes such as Upper Mustang, where direct observations are rare and ecological baselines remain poorly established.

In this study, we present the first genetically confirmed record of the Tibetan brown bear ( *Ursus arctos pruinosus* ) from central Nepal, based on mitochondrial haplotype analysis of non‐invasively collected samples, as well as multi‐year camera‐trap data from the Upper Mustang region of the Annapurna Conservation Area. By combining genetic evidence with camera‐trap detections as part of a broader mammalian community survey, we (i) confirm the subspecific identity of brown bears occurring in central Nepal, (ii) document their occurrence within a high‐altitude predator–prey assemblage, and (iii) provide critical baseline information to inform future monitoring and conservation planning for one of Nepal's most threatened large carnivoran.

## 2 Materials and Methods

### 2.1 Study Area

The study was conducted in the Trans‐Himalayan region of western Nepal, encompassing Mustang District (3573 km^2^) within the Annapurna Conservation Area (ACA; 7629 km^2^) (NTNC 2012) and Dolpa District (7889 km^2^) within Shey–Phoksundo National Park (SPNP; 3555 km^2^) (SPNP 2023) (Figure 1). Both regions are characterized by cold arid climates, rugged mountainous terrain, and alpine steppe vegetation, and provide important habitats for high‐altitude carnivores such as the brown bear ( *Ursus arctos* ), snow leopard ( *Panthera uncia* ), and Himalayan wolf ( *Canis lupus chanco* ) (Acharya and Paudel 2020; Aryal, Hopkins, et al. 2012; Baral et al. 2024; Chetri 2022; Lamichhane et al. 2025; SPNP 2023).

**FIGURE 1:**
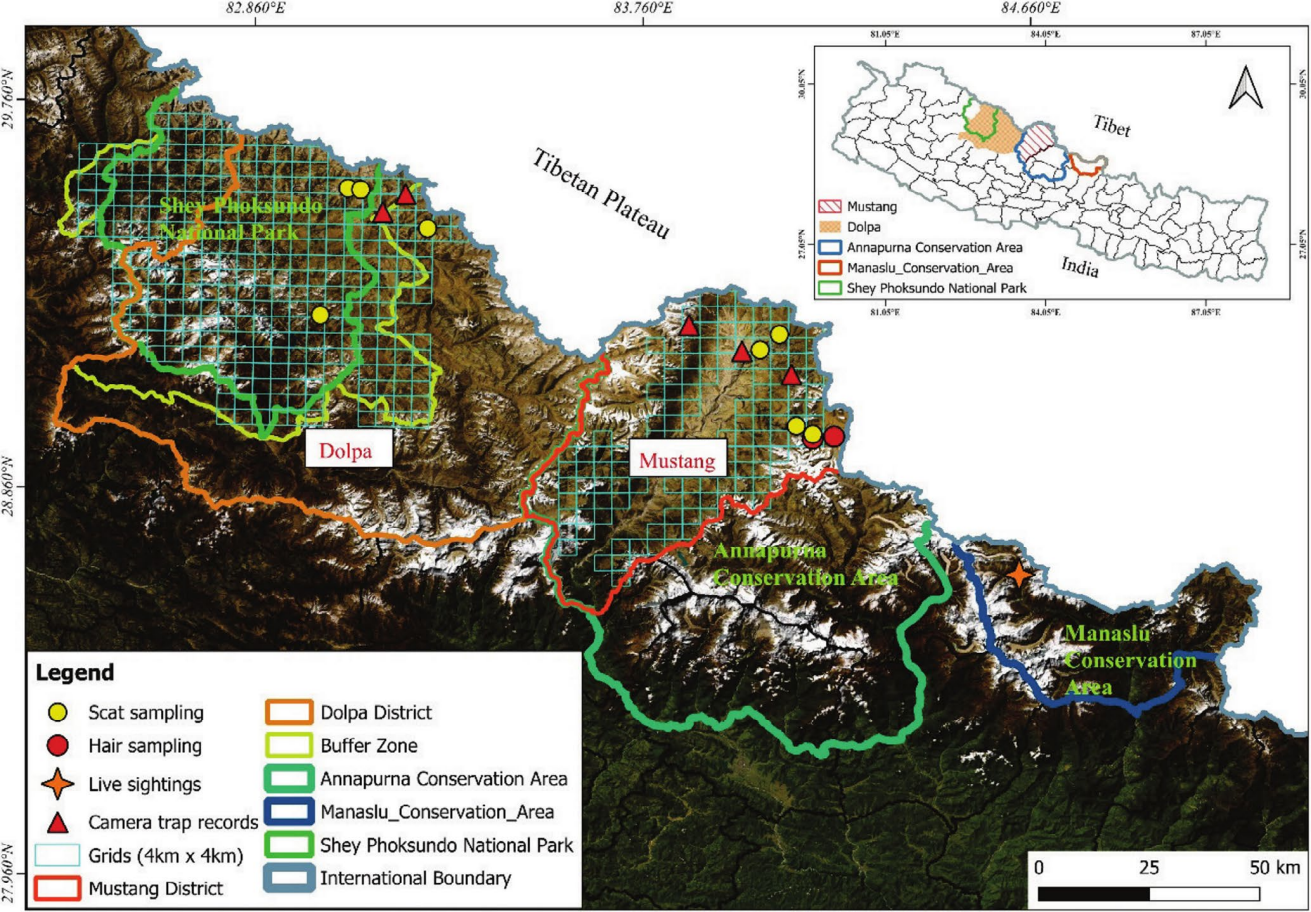
Study area map showing the sampling site and camera‐trap location. Yellow circles are of scat sample collection sites, red circles denote hair samples collection sites, red triangles indicate brown bear camera‐trap records, and the one orange star is the location of the live sighting observation. Records were compiled from published literature, technical reports, and the present study, covering the period from 2008 to 2025.

Non‐invasive genetic samples were collected from both Mustang and Dolpa districts, whereas camera‐trap surveys were conducted exclusively within the ACA, exclusively on the Upper Mustang region. Elevations across the sampled areas ranged from approximately 3500 to 6000 m *a.s.l*., sites consistent with the Trans‐Himalayan cold‐desert environment (Aryal 2013; Chetri and Gurung 2004).

### 2.2 Genetic Sample Collection

Non‐invasive genetic samples were collected opportunistically during field surveys conducted between 2022 and 2024 in Mustang District (ACA) and Dolpa District (SPNP). Samples consisted primarily of putative brown bear feces, with occasional hair samples, located using associated field signs such as tracks, digging marks, and feeding sites (Aryal, Hopkins, et al. 2012; Nawaz 2008). We collected samples with the help of experienced local field personnel familiar with wildlife sign in the region.

For fecal samples, we “swabbed” the outer mucosal layer using sterile swabs following established non‐invasive sampling protocols (De Barba et al. 2010; Paudel et al. 2026; Taberlet et al. 1997) and preserved in InhibitEX buffer (Qiagen, Germany). All hair samples were collected using sterilized forceps and stored in paper envelopes (Taberlet et al. 1999). We used disposable gloves for all samples, changing between samples, and all tools and supplies were sterilized with ethanol to prevent cross‐contamination. Finally, we geo‐referenced all samples using a handheld GPS and stored them at −20°C prior to subsequent laboratory analysis.

### 2.3 DNA Extraction and Molecular Analyses

Under sterile laboratory conditions, we extracted DNA from fecal samples and isolated it using the QIAamp DNA Stool Mini Kit (Qiagen, Germany). In addition, we extracted DNA from hair samples using the Isohair Easy Kit (Nippon Gene, Japan) and followed the manufacturer's protocols. We also included negative extraction controls to monitor for contamination (De Barba et al. 2010; Taberlet et al. 1996). To identify subspecies, we amplified a short fragment of the mitochondrial control region (D‐loop) using bear‐specific primers previously described by Lan et al. (2017). For PCR amplification and sequencing, we followed the protocols of Matsuhashi et al. (1999) for degraded DNA recovered from non‐invasive samples. Sequences were then edited and aligned using MEGA v12 (Kumar et al. 2024).

Phylogenetic relationships were inferred using Maximum Likelihood methods under the Kimura 2‐parameter model (Kimura 1980), with node support assessed through 1000 bootstrap replicates (Saitou and Nei 1987). Subspecies identity was determined based on sequence similarity and phylogenetic clustering with reference haplotypes of *Ursus arctos pruinosus* and *U. a. isabellinus* as retrieved from GenBank (Galbreath et al. 2007; Lan et al. 2017). Microsatellite genotyping was used to identify unique individuals and avoid duplicate sampling; however, subspecies‐level assignment relied primarily on mitochondrial DNA evidence (Paetkau et al. 1995; Shimozuru et al. 2022). Detailed laboratory protocols, including microsatellite loci, PCR reaction volumes, thermal cycling conditions, sequencing chemistry, and genetic data processing steps, are provided in Supporting Information S1.

### 2.4 Camera‐Trap Survey

We conducted camera‐trapping primarily within the Annapurna Conservation Area (ACA) in the Upper Mustang region between September 2022 and June 2024. The camera‐trapping was conducted as part of a broader survey of the resident mammalian community. To complement these data, camera‐trap records from Dolpa District, collected during separate survey periods but following the same survey protocol, were also included to document brown bear occurrence. Camera‐trap deployment occurred over multiple field sessions, including installation (September–October 2022), maintenance and data retrieval (April–May 2023 and October–November 2023), and additional deployments (May–June 2024).

We used a systematic 4 × 4 km^2^ grid‐based design to ensure spatial coverage and independence of detection events following previously‐established large carnivore survey protocols (Jackson and Lama 2016; O'Connell et al. 2011). In Upper Mustang, 74 grid cells were identified within the elevation range of 3500–5600 m a.s.l., covering Lomanthang and Lo‐Ghekar Damodarkunda Rural Municipalities. In Dolpa District, 32 grid cells were identified within comparable high‐altitude habitats. Camera stations were not deployed in every grid cell; instead, locations were selected opportunistically within selected grid cells based on field evidence of brown bear and other large mammal activity, including animal signs, trails, ridgelines, valley bottoms, and natural movement corridors. To reduce spatial dependence among detections, adjacent camera stations were separated by a minimum distance of 2 km. Camera stations remained fixed at their selected locations throughout the survey period and were not rotated among grid cells. No visual or olfactory attractants (bait or scent lures) were used during camera‐trapping.

A total of 96 camera‐trap stations were deployed during the study, comprising 64 stations in Mustang and 32 stations in Dolpa. Camera trapping in both study areas followed the same survey protocol. The Mustang camera traps were established specifically for this study, whereas the Dolpa camera traps were part of a long‐term snow leopard monitoring program from which brown bear records were obtained opportunistically. Each camera‐trap station consisted of a single motion‐triggered infrared camera mounted approximately 40–50 cm above ground level and programmed to operate continuously. Cameras were placed along wildlife trails, ridgelines, valley bottoms, and natural movement corridors identified through field sign surveys and prior knowledge of large mammal movements (Burton et al. 2015; Chetri et al. 2017).

In addition to the Upper Mustang survey, supplementary camera‐trap data from Dolpa district (western Nepal) were included to document incidental records of brown bear occurrence. Camera‐trapping in Dolpa was conducted as part of long‐term snow leopard research during two survey periods: 3 March–25 Oct 2024 and 3 March–25 June 2023. Although conducted during different time periods from the Upper Mustang survey, the Dolpa camera trapping followed the same sampling protocol, including camera deployment strategy, placement criteria, and survey design. Although brown bears were not the intended target species, our camera‐traps did record brown bear presence in high‐altitude alpine habitats. These records, however, were treated as opportunistic detections and were ultimately not included in estimates of community‐wide sampling effort or detection frequency for Upper Mustang.

All images were screened to identify species, and then we extracted temporal (date) and spatial (location) metadata. Brown bears were initially differentiated from black bears and other species (especially at a distance) based on diagnostic morphological characteristics; these include large body size, pelage coloration, prominent shoulder hump, and concave facial profile, following established descriptions of *Ursus arctos* (McLellan et al. 2017; Nawaz 2008). To confirm subspecific identity across both study areas (Upper Mustang and Dolpa), relevant camera‐trap images were independently reviewed by experts from the IUCN Species Survival Commission (SSC) Bear Specialist Group (BSG).

## 3 Results

### 3.1 Genetic Sample Recovery and Amplification Success

We ultimately collected a total of 10 non‐invasive genetic samples putatively attributed to brown bears from Upper Mustang (*n* = 6) and Dolpa District (*n* = 4) between 2022 and 2024 (Table 1). These included seven fecal samples and three hair samples. Of these, seven samples yielded successful mitochondrial DNA sequences; four were successfully genotyped using microsatellite markers.

**TABLE 1:** Summary of microsatellite genotyping results for brown bear samples collected from Dolpa and Mustang, Nepal.

Sample ID	District	Sample type	Sex	mtDNA haplotype	Bear ID	Genotype status
2023S1	Mustang	Feces	NA	NA	—	Failed
2022S2	Mustang	Feces	Male	Tibetan	—	Failed
22CH01	Mustang	Hair	Male	Tibetan	A	Successful
22DH04	Mustang	Hair	NA	NA	—	Failed
22DS01	Mustang	Hair	NA	Tibetan	—	Failed
22DS02	Mustang	Feces	NA	Tibetan	—	Failed
TN19	Dolpa	Feces	Male	Tibetan	B	Successful
Sd70Ly	Dolpa	Feces	Male	NA	B	Successful
Sd70	Dolpa	Feces	Male	Tibetan	B	Successful
PH76	Dolpa	Feces	NA	Tibetan	—	Failed

Abbreviation: NA, not available.

Microsatellite genotyping also helped us identify two unique individuals. One individual (Individual A) was detected in Upper Mustang from a single hair sample (22CH01), whereas a second individual (Individual B) was identified from three fecal samples collected in Dolpa (TN19, Sd70, Sd70Ly); these individuals shared identical genotypes (Supporting Information Tables S2 and S3). All successfully genotyped individuals were identified as males. The overall microsatellite genotyping success rate was 40% (4/10 samples), with successful genotypes obtained from one of three hair samples (33.3%) and three of seven fecal samples (42.9%).

### 3.2 Mitochondrial Haplotype and Subspecies Identification

Mitochondrial control‐region sequences that we obtained from all successfully amplified samples were consistent with a single haplotype shared by individuals from both Mustang and Dolpa. Haplotype diversity and nucleotide diversity were both zero, indicating no detectable mtDNA variation among the samples we analyzed.

Our phylogenetic analysis suggested all Nepalese brown bear sequences are from within the Tibetan brown bear lineage ( *Ursus arctos pruinosus* ), as they formed a well‐supported clade with reference sequences originating from the Tibetan Plateau (Figure 2). The Himalayan brown bear (*U. a. isabellinus*) formed a distinct sister lineage, clearly distinct from the Nepalese samples. Other brown bear lineages also formed geographically distinct clades, and we used the sloth bear ( *Melursus ursinus* ) as our divergent outgroup. Bootstrap support values for major nodes were > 70%, indicating strong phylogenetic support for these conclusions.

**FIGURE 2:**
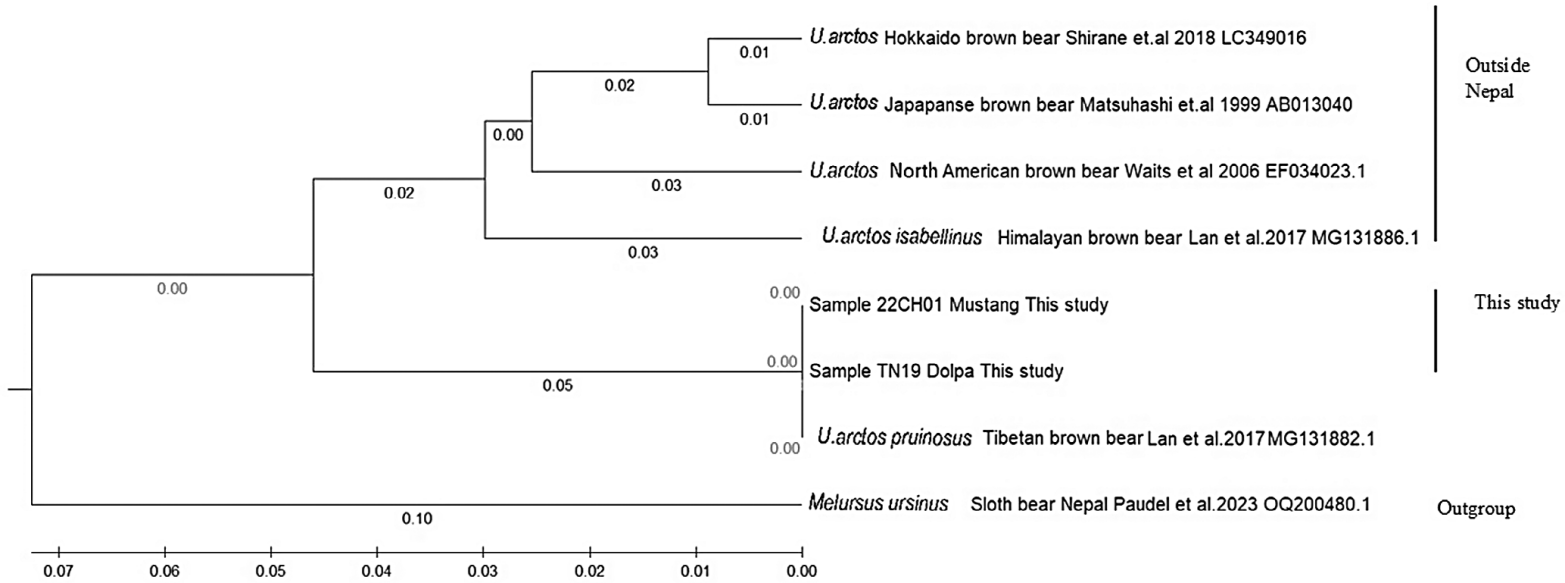
Phylogenetic relationships of brown bears including samples from Nepal based on mitochondrial DNA sequences. Numbers on branches indicate genetic distances (substitutions per site), representing the amount of nucleotide divergence between lineages. Nepalese samples show zero or near‐zero genetic distance from *U. arctos pruinosus* , indicating identical or nearly identical haplotypes. *Melursus ursinus* was used as an outgroup.

### 3.3 Camera‐Trap Evidence of Brown Bear Presence in Upper Mustang and Dolpa, Nepal

Of the 96 camera‐trap stations deployed (64 in Mustang and 32 in Dolpa), mammals were detected at 39 stations in Mustang and 18 stations in Dolpa, representing a total sampling effort of 9336 camera‐trap nights (6506 in Mustang and 2830 in Dolpa). All brown bear detections in Upper Mustang occurred at elevations ranging from 3834 to 4,816 m *a.s.l*. within the ACA; they were found across alpine steppe, shrubland, and grassland habitats (Table 2; Figure 1).

**TABLE 2:** Brown bear camera‐trap records in Mustang and Dolpa, Nepal.

Capture date	Site/Block	Coordinates (°N, °E)	Elevation (m)	Habitat	Time(s) of capture	Observed behavior(s)
30‐Apr‐23	Lomanthang/Mustang	29.17443, 83.98973	3834	Alpine steppe	06:33; 17:58	Walking
5‐Jun‐23	Damodar Kunda/Mustang	29.12068, 84.10466	4280	Shrubland	21:08; 21:17	Playing near camera; walking
20‐Jun‐24	Lomanthang/Mustang	29.20602, 83.88190	4816	Grassland	8:24	Walking
2‐May‐2023	Laru/Dolpa	29.53989, 83.2088	4940	Alpine steppe	7.05	Walking/looking at camera trap
1‐ August 2024, 3‐Sept 2024, 15‐Sept 2024	Chiyol, Musi village/Dolpa	29.49751, 83.15547	4167	Alpine steppe	04.02, 20.52, 03.15	Walking/looking at camera trap

Using a criterion of detections separated by at least 30 min as independent capture events, we recorded four independent brown bear capture events at three camera‐trap stations in Upper Mustang between April 2023 and June 2024 (Table 2; Figure 3). On 30 April 2023 at Lomanthang, we detected a brown bear twice on the same day: the first was during the early morning (06:33 h) while moving upslope, and another time during the late evening (17:58 h), while moving downslope (Figures 3a, 2b). During the morning, we also detected five feral dogs in the vicinity, which suggested that the bear may have concealed itself during daylight hours and resumed movement later in the evening. On 5 June 2023, we logged two consecutive detections at night at Damodar Kunda (21:08 h and 21:17 h), which were considered a single independent capture event because they occurred within 30 min, where the bear was observed playing and repeatedly moving near the camera trap, including jumping in front of the camera (Figure 3c). We also recorded a single early‐morning detection on 20 June 2024 at Lomanthang (08:24 h), showing the bear walking calmly in front of the camera trap (Figure 3d).

**FIGURE 3:**
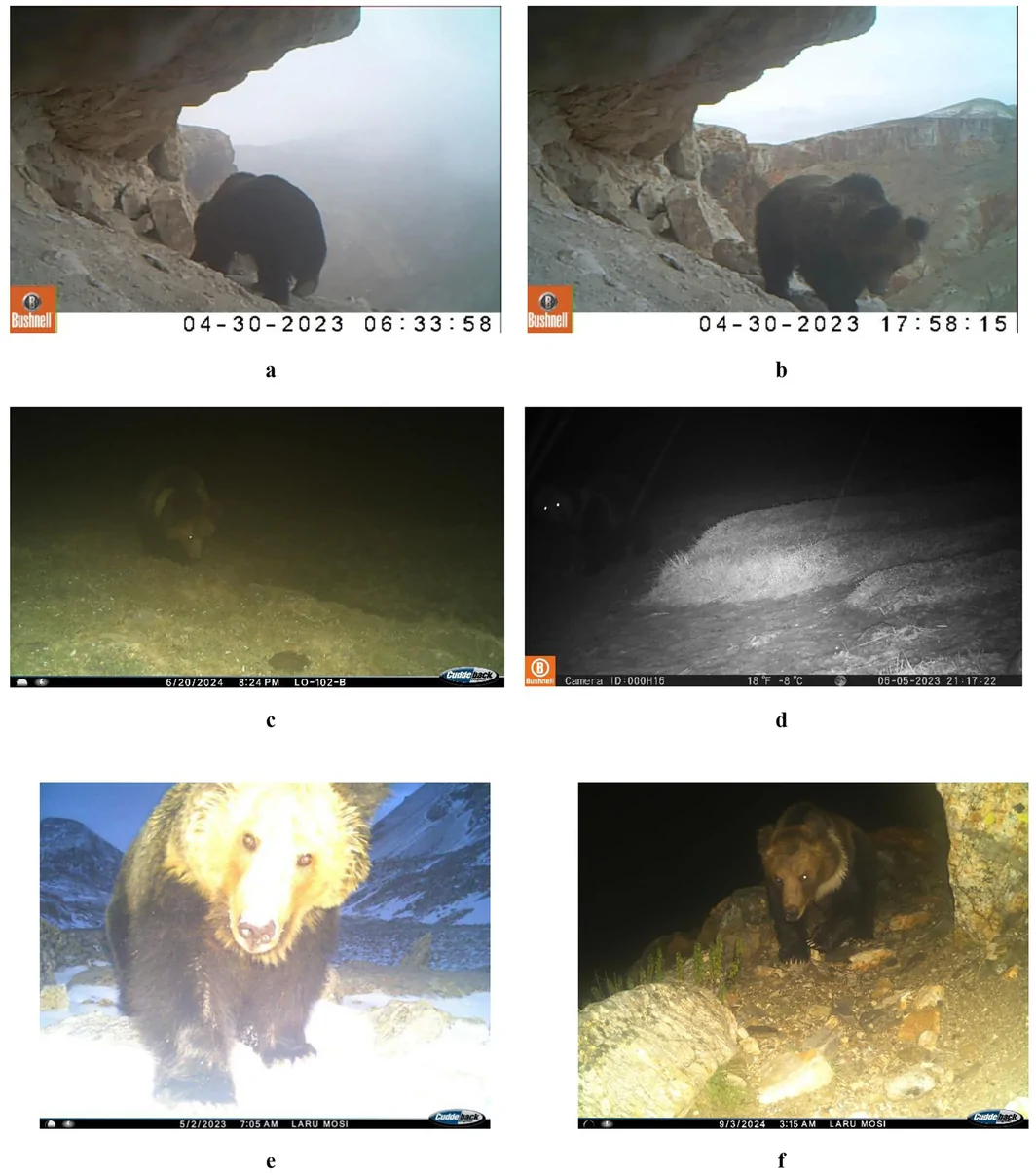
Camera‐trap images of Tibetan brown bears recorded in the Dolpa and Upper Mustang region between 2023 and 2024: (a–b) repeated detections at Lomanthang on 30 April 2023; (c) nocturnal activity at Damodar Kunda on 5 June 2023; (d) early‐morning detection at Lomanthang on 20 June 2024; (e) morning detection at Laru, Mosi village, Dolpa on 2 May 2023; (f) detections at Chiyol, Musi village, Dolpa on 3 September, 2024.

In addition to Upper Mustang, we also obtained camera‐trap evidence of brown bears from Dolpa district in western Nepal. We conducted camera‐trapping in Dolpa as part of a long‐term snow leopard ( *Panthera uncia* ) research project during two survey periods: 3 March to 25 June 2023, and 3 March to 25 Oct 2024. During these surveys, Dolpa camera‐trapping consisted of 32 camera‐trap stations, generating incidental records of brown bear occurrence in high‐altitude alpine habitats. Although brown bears were not our target species, these incidental detections occurred in high‐altitude alpine habitats. Three brown bear detections were recorded at Chiyol, Musi village, Dolpa (29.49751°N, 83.15547°E), on 1 August, 3 September, and 15 September 2024, at an elevation of 4167 m a.s.l. within alpine steppe habitat. We also recorded one additional detection on 2 May 2023 at Laru, Musi village, Dolpa (29.53989°N, 83.20880°E) at an elevation of 4940 m *a.s.l*., also in alpine steppe habitat (Table 2; Figure 3e). The individual was observed walking and briefly inspecting the camera trap during the morning hours (07:05 h).

Across both Upper Mustang and Dolpa, suspected brown bear images consistently showed a large bear‐bodied bear with pale to dark brown pelage, a prominent shoulder hump, and a concave facial profile—all diagnostic morphological characteristics of the brown bear ( *Ursus arctos* ). These photographic records provided clear visual confirmation of brown bear occurrence in central and western Nepal. Confirmation of subspecies was further corroborated through expert‐based morphological assessments. Relevant camera‐trap images were independently reviewed by experts from the IUCN Species Survival Commission (SSC) Bear Specialist Group (BSG). Based on these diagnostic morphological features, including pelage coloration and texture, overall body proportions, and craniofacial profile, experts consistently identified the recorded bears from Upper Mustang and Dolpa as consistent with the Tibetan brown bear ( *Ursus arctos pruinosus* ).

### 3.4 Additional Live Sighting Record

In addition to the camera‐trap records we describe herein, we obtained a verifiable photographic record of a live brown bear from a location above a hill near Samdo village (28.6583321°N, 84.6486364°E) in the Manaslu Conservation Area, central Nepal (Figure 4). The photograph was taken by a local horse owner using a mobile phone while he was searching for his horse in nearby grazing land. The observer initially noticed two Himalayan vultures ( *Gyps himalayensis* ) at the site and subsequently detected the brown bear in the same area.

**FIGURE 4:**
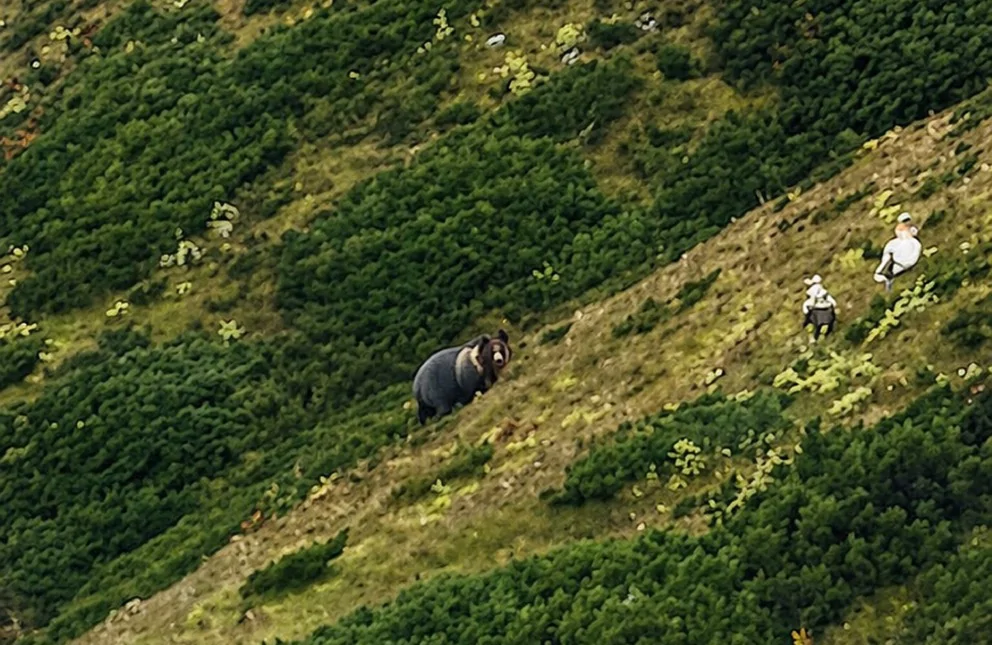
Verified live photographic record of a Tibetan brown bear from the Manaslu Conservation Area, central Nepal. Photo by Rinjin Dhorje Lama.

The image shows a brown bear feeding on a carcass, indicative of either scavenging behaviuor or possible predation on a large mammal. This individual exhibits morphological characteristics consistent with the Tibetan brown bear ( *Ursus arctos pruinosus* ), including large body size, pelage coloration, and a prominent shoulder profile.

This additional record provides supplementary evidence of brown bear occurrence in central Nepal, extending other confirmed records beyond our camera‐trap and genetic data, and indicating the continued presence of the species in the Trans‐Himalayan landscapes of the country.

### 3.5 Spatial Distribution of Brown Bear Records in Nepal

We synthesized all of our data, including camera‐trap detections, genetically confirmed samples, verified personal observations of live animals, and records obtained from published studies and technical reports, to map the current distribution of brown bear records in Nepal (Figure 5). These compiled records span from 2008 to 2025, beginning with the first published record of brown bears from the Upper Mustang region (Chetri 2008), and represent the currently known distribution of brown bears across Nepal. Our study documented the occurrence of brown bears from multiple Trans‐Himalayan landscapes, including the Limi Valley (Humla District), Shey Phoksundo National Park (Dolpa District), the Upper Mustang region of the Annapurna Conservation Area (ACA) (Mustang District), Samdo village in the Manaslu Conservation Area (Gorkha District), and Yangma in the Kanchenjunga Conservation Area (Ilam District). When viewed in the context of the IUCN Red List Assessment global distribution range of *Ursus arctos* (McLellan et al. 2017), our confirmed records from Nepal fall predominantly within the central and eastern Himalayan portion of the bear's range (Figure 5). Collectively, however, these records demonstrate that brown bears occur across several Trans‐Himalayan landscapes of central and western Nepal. These regions also all appear to represent ecologically suitable landscapes for brown bears within Nepal.

**FIGURE 5:**
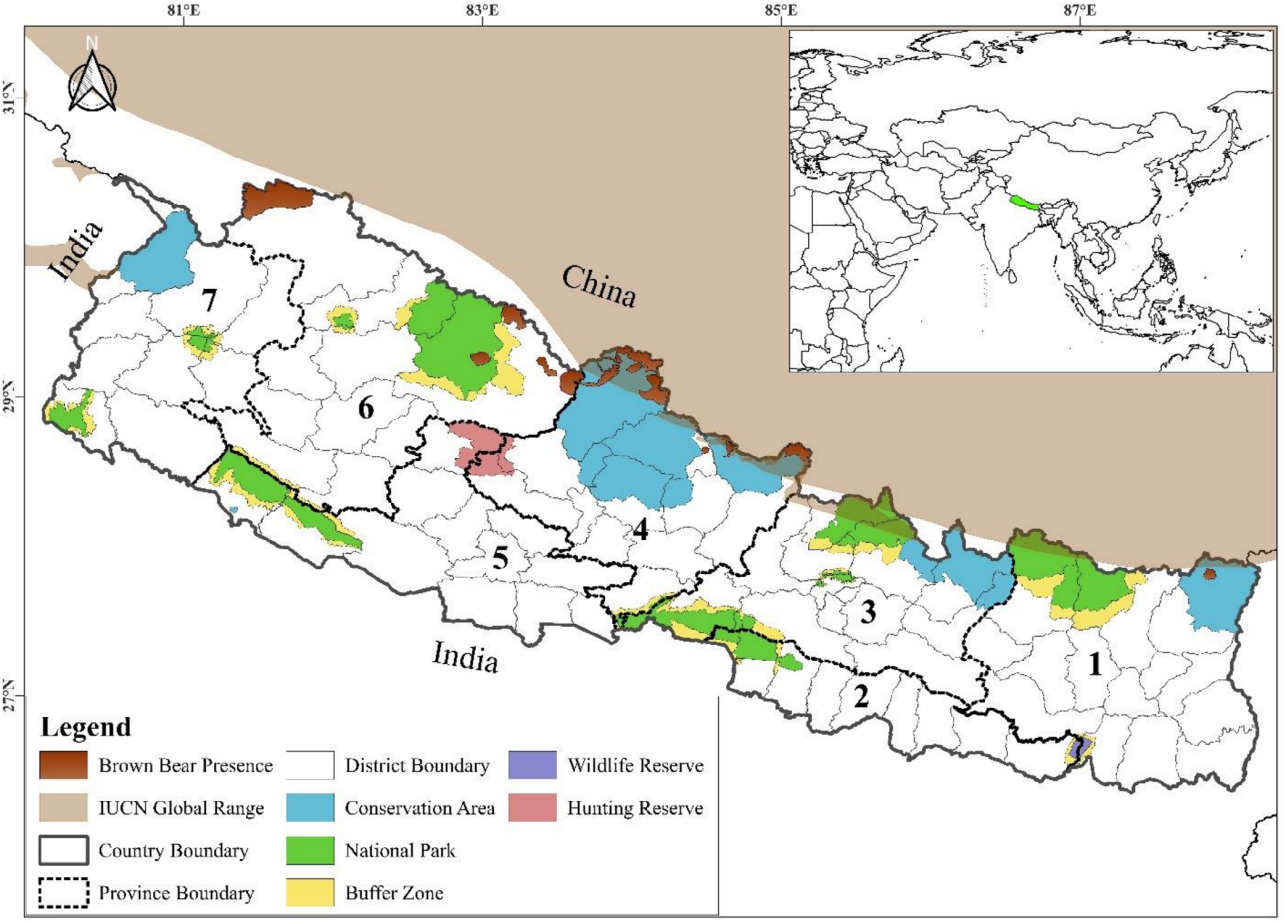
Current distribution of confirmed brown bear ( *Ursus arctos* ) records in Nepal in relation to the IUCN global distribution range. Records were compiled from published studies, technical reports, and the present study, covering the period from 2008 to 2024. Province boundaries are shown for reference: (1) Koshi Province, (2) Madhesh Province, (3) Bagmati Province, (4) Gandaki Province, (5) Lumbini Province, (6) Karnali Province, and (7) Sudurpashchim Province.

## 4 Discussion

### 4.1 Genetic Confirmation of the Tibetan Brown Bear in Nepal

Our study provides the first genetically confirmed evidence of the Tibetan brown bear ( *Ursus arctos pruinosus* ) in Nepal. Our phylogenetic analyses unequivocally placed all Nepalese samples within the Tibetan brown bear lineage, one that is clearly separate from the Himalayan brown bear (*U. a. isabellinus*) lineage, with strong bootstrap support. This genetic confirmation resolves long‐standing uncertainty regarding the identity of the brown bear subspecies inhabiting Nepal's Trans‐Himalayan region.

Prior to this study, no previous study had been conducted on the genetics of brown bears within Nepal. Existing knowledge of brown bear occurrence in the country has relied primarily on indirect signs, anecdotal camera‐trap images, habitat modeling with sparse data, and opportunistic sightings (Aryal, Raubenheimer, et al. 2012; Chetri 2022; Kusi et al. 2024). While these studies ultimately expanded the known distribution of brown bears in Nepal, they were unable to distinguish subspecies due to overlapping morphological characteristics and the absence of molecular data.

However, genetic studies of brown bears from surrounding regions provide us with additional important context. Mitochondrial and nuclear DNA analyses from the Tibetan Plateau, Qinghai–Tibetan Plateau, for example, and adjacent regions of China, consistently identify Tibetan brown bears (*U. a. pruinosus*) as a genetically distinct lineage, separate from the Himalayan brown bear (*U. a. isabellinus*) of Pakistan and northwestern India (Galbreath et al. 2007; Lan et al. 2017; Yu et al. 2007). Our findings demonstrate that brown bears inhabiting Nepal's Trans‐Himalayan landscapes are genetically aligned with the Tibetan Plateau lineage rather than with Himalayan brown bears, thereby confirming the occurrence of *U. a. pruinosus* at the southern margin of its known distribution. By providing the first molecular evidence from Nepal, this study fills a critical geographic and taxonomic gap in brown bear phylogeography across the Himalayas, and resolves ambiguity surrounding subspecies boundaries at the southern edge of the Tibetan Plateau.

### 4.2 Genetic Diversity, Population Isolation, and Demographic Implications

All successfully amplified mitochondrial sequences from Mustang and Dolpa shared a single haplotype, resulting in zero haplotype and nucleotide diversity. While this result should be interpreted with caution due to our limited sample size, similar patterns of low mtDNA diversity have been widely reported for Tibetan brown bear populations across their core range. Studies from the Qinghai–Tibetan Plateau and adjacent regions, for example, have documented reduced mtDNA variation, which has often been attributed to historical bottlenecks, long‐term isolation, and restricted female‐mediated dispersal in these extreme high‐altitude environments (Buono et al. 2022; Lan et al. 2017; Yu et al. 2007).

The relatively low microsatellite amplification success in this study (40%; 4/10 samples) represents an important limitation. Although hair samples can often provide high‐quality nuclear DNA when intact follicles are available, amplification success depends strongly on sample quality, DNA quantity, preservation, and laboratory procedures (Taberlet et al. 1997; Taberlet et al. 1999; Waits and Paetkau 2005). In this study, only one of three hair samples produced a successful genotype (33.3%). This lower success may have resulted from variation in the amount of follicular tissue available, environmental degradation before sample collection, or prolonged exposure to harsh Trans‐Himalayan conditions, including intense solar radiation, low humidity, and large temperature fluctuations. Non‐invasive samples collected under field conditions can experience DNA degradation, leading to reduced amplification success and increased genotyping failure rates (Taberlet et al. 1999; Waits and Paetkau 2005). Furthermore, the limited number of available samples reduced opportunities for repeated amplification and genotype validation. Therefore, although our findings provide the first molecular confirmation of Tibetan brown bears in Nepal, conclusions regarding population‐level genetic diversity, abundance, and connectivity should be interpreted cautiously until larger datasets using additional nuclear markers become available.

Low mtDNA diversity is not unique to Tibetan brown bears, and has also been reported in peripheral or isolated brown bear populations from Europe, Central Asia, and North America, particularly those in small or fragmented populations (Galbreath et al. 2007; Kutschera et al. 2014; Zemanova 2021). These patterns are also commonly associated with reduced effective population sizes and limited connectivity, which may increase vulnerability to demographic and environmental stochasticity.

The Trans‐Himalayan landscapes of Nepal are characterized by steep topography, glaciated passes, arid valleys, and spatially fragmented alpine habitats, which likely further constrain gene flow among local bear populations (Hirata et al. 2013; Itoh et al. 2012). The identification of only male individuals in this study is consistent with findings from other brown bear genetic surveys, where males are more frequently detected due to larger home ranges and greater dispersal tendencies, whereas females exhibit strong philopatry (Waits and Paetkau 2005; Zemanova 2021). This sex‐biased detectability suggests that female‐mediated gene flow may be particularly limited in Nepal, potentially exacerbating genetic isolation or seasonal migration from Tibet.

### 4.3 Camera‐Trap Evidence in Relation to Genetic Findings

Our camera‐trap records from Upper Mustang provide independent ecological corroboration of our genetic data confirming the presence of Tibetan brown bears in central Nepal. Four capture events recorded between 2023 and 2024 at three stations documented bear activity across alpine steppe, shrubland, and grassland habitats between 3834 and 4816 *m a.s.l*. These habitat types and elevation ranges closely match those reported for Tibetan brown bears elsewhere on the Tibetan Plateau, reinforcing the genetic identification (Dai et al. 2019; Lan et al. 2017).

Observed behaviors, including nocturnal activity, localized movement, and apparent avoidance of domestic dogs, are consistent with brown bear ecology in human‐modified alpine landscapes (Dai et al. 2019; Nawaz 2008). The integration of camera traps and genetic data thus strengthens our confidence that photographic records from Upper Mustang and Dolpa represent *U. a. pruinosus* rather than morphologically similar Himalayan brown bears.

### 4.4 Distributional Implications and Peripheral Populations

When integrated, genetic records from Dolpa and Upper Mustang, and camera‐trap detections and verified sightings from Limi Valley, Manaslu Conservation Area, and eastern Nepal, suggest that Tibetan brown bears persist across a network of isolated pockets in the Trans‐Himalayan landscapes in Nepal. However, when viewed in relation to the global distribution of *Ursus arctos* defined by the IUCN, all Nepalese records occur along the southern periphery of the Tibetan Plateau population, highlighting their status as marginal or peripheral populations at the edge of their range.

Peripheral populations often exhibit reduced genetic diversity and heightened vulnerability to environmental change, human disturbance, and demographic stochasticity (Buono et al. 2022; Galbreath et al. 2007). The Nepalese Tibetan brown bear populations likely represent small, semi‐isolated extensions of the main Tibetan Plateau population, which could align with important stepping stone or refugial habitats under future climate change scenarios. However, the fragmented nature of suitable habitat in Nepal, combined with increasing infrastructure development, livestock grazing, and human–bear conflict, may further threaten the persistence of these small and isolated populations (Aryal et al. 2014; Dai et al. 2019).

### 4.5 Conservation Significance

The genetic confirmation of *Ursus arctos pruinosus* in Nepal provides important baseline information for understanding the distribution and conservation status of Tibetan brown bears at the southern margin of their known range. As a nationally Critically Endangered taxon, Tibetan brown bears require conservation strategies tailored to their restricted distribution, low genetic diversity, and fragmented habitat. However, given the limited number of genetically confirmed individuals in this study, our findings should be interpreted cautiously. Our data confirm the presence of at least two male Tibetan brown bears in Nepal but do not provide direct evidence of a resident breeding population. Because male brown bears generally exhibit larger home ranges and greater dispersal tendencies than females (Waits and Paetkau 2005; Zemanova 2021), it is possible that some individuals detected in Nepal may represent dispersing or transient individuals from adjacent populations on the Tibetan Plateau. Therefore, further research is required to determine whether Nepal supports resident populations, seasonal migrants, or movement corridors connecting populations across the Tibetan Plateau and Himalayan regions.

Nevertheless, repeated brown bear records across multiple years and locations, including Mustang, Dolpa, Humla, Manaslu, and eastern Nepal, suggest that Nepal's Trans‐Himalayan landscapes are regularly used by brown bears rather than representing isolated accidental observations. The absence of detectable mitochondrial variation and our evidence of population isolation highlight the urgency of maintaining landscape connectivity across Trans‐Himalayan corridors, including the reduction of anthropogenic impacts.

If Tibetan brown bears are confirmed to maintain resident populations and reproduce within Nepal, conservation priorities should include protection of critical alpine habitats, maintaining landscape connectivity, and mitigating human–bear conflict in pastoral landscapes. Conversely, if Nepal primarily functions as a seasonal movement corridor or peripheral habitat for individuals originating from larger Tibetan Plateau populations, conservation efforts would remain important for maintaining habitat connectivity and supporting population exchange.

Future conservation and research priorities might include expanded non‐invasive genetic sampling across additional regions, particularly targeting females and reproductive evidence, the incorporation of nuclear genomic markers to assess connectivity and effective population size, and mitigation of human–bear conflict in pastoral landscapes. This study establishes the first molecular baseline for Tibetan brown bears in Nepal and provides an initial foundation for future conservation planning. It also emphasizes the importance of integrating multiple methodologies, including genetics, camera trapping, and local ecological knowledge, to study an elusive species with large spatial requirements. Long‐term monitoring will be necessary to clarify population size, breeding status, and the degree of connectivity between Nepalese and Tibetan Plateau populations. Transboundary collaboration with neighboring regions of the Tibetan Plateau, particularly research and conservation professionals in China, will be essential to ensure the long‐term persistence of this globally rare subspecies in Nepal.

## Author Contributions

**Rishi Baral:** conceptualization (lead), data curation (lead), formal analysis (lead), funding acquisition (lead), investigation (lead), methodology (lead), project administration (equal), resources (lead), software (equal), validation (lead), visualization (lead), writing – original draft (lead), writing – review and editing (lead). **Ram Bahadur Gurung:** conceptualization (supporting). **Abhisek Sapkota:** conceptualization (supporting), writing – review and editing (supporting). **Naresh Subedi:** conceptualization (supporting), supervision (supporting), writing – review and editing (supporting). **Anthony J. Giordano:** conceptualization (supporting), writing – review and editing (supporting). **Rajan Prasad Paudel:** conceptualization (supporting), data curation (supporting). **Rabin Kadariya:** conceptualization (supporting), resources (supporting). **Gopal Khanal:** conceptualization (supporting), project administration (supporting), resources (supporting). **Kinoshita Kodzue:** conceptualization (supporting), funding acquisition (supporting), methodology (supporting), project administration (supporting). **Toshio Tsubota:** conceptualization (equal), funding acquisition (equal), investigation (equal), supervision (lead). **Michito Shimozuru:** conceptualization (equal), formal analysis (supporting), methodology (supporting), supervision (equal), validation (supporting).

## Funding

This research was supported by the International Association for Bear Research and Management through a Research and Conservation Grant (IBA‐SG_2022‐23_03). The first author (Rishi Baral) received doctoral funding from Hokkaido University, including support from the Hokkaido University, EXEX Doctoral Fellowship, Grants‐in‐Aid for Scientific Research, and the WISE Program. Field logistics were supported by the Annapurna Conservation Area Project (ACAP) under the National Trust for Nature Conservation (NTNC). Kinoshita Kodzue was supported by the JSPS KAKENHI Grant‐in‐Aid for Scientific Research (B) (20H03008, 24K03130) and the Japan Prize Foundation (Gopal Khanal) was supported by Japan Fund for Global Environment and Nagao Natural Environment Foundation. Toshio Tsubota was supported by the Asahi Glass Foundation (Grant No. 2021‐94) and Hokkaido University Crowdfunding (2023–2024).

## Ethics Statement

All field surveys and biological sample collection were conducted under permits issued by the Department of National Parks and Wildlife Conservation (DNPWC), Nepal, and the National Trust for Nature Conservation/Annapurna Conservation Area Project (NTNC/ACAP). All methods followed national wildlife research regulations, and only non‐invasive techniques were employed (Government of Nepal 1973).

## Conflicts of Interest

The authors declare no conflicts of interest.

## Supporting information


**Supporting Information: S1** Detailed laboratory protocols for genetic analyses.

**Table S2:** Database of two Tibetan brown bears for 8 microsatellite loci for two Tibetan brown bears in Nepal.

**Table S3:** Aligned DNA sequence.

## Data Availability

The data that supports the findings of this study are available in the Supporting Information of this article.
