# Supplementary material for: Confirmation of the Tibetan Brown Bear (Ursus arctos pruinosus) in Central Nepal Based on Genetic and Camera‐Trap Evidence
Source: Ecol Evol. 2026 Oct 7;16(10):e74400. doi: 10.1002/ece3.74400 (PMC13645494; doi:10.1002/ece3.74400)
Supplement: Supplementary file 1 [file ECE3-16-e74400-s001.docx]

**Supplementary Material S1. Detailed laboratory protocols for genetic analyses**

**DNA extraction**

Genomic DNA was extracted from non-invasive fecal and hair samples under sterile laboratory conditions to minimize contamination risk. All samples were stored at −20 °C prior to extraction. DNA from fecal samples was extracted using the QIAamp DNA Stool Mini Kit (Qiagen, Germany), while DNA from hair samples was extracted using the Isohair Easy Kit (Nippon Gene, Tokyo, Japan), following the manufacturers’ instructions. Negative extraction controls were included in each batch to monitor potential contamination.

**Microsatellite genotyping**

Microsatellite genotyping was conducted using eight loci commonly applied in brown bear studies: G1A, MU05, MU51, MU50, G10B, MU23, UamD2, and UamB5, originally developed by Paetkau et al. (1995, 1997), Taberlet et al. (1997), Andreassen et al. (2012), and De Barba et al. (2010). A sex marker (SE47/48) was included following Yamamoto et al. (2002), consistent with previously established protocols for brown bears (Shimozuru et al., 2022; Shirane et al., 2018).

PCR amplifications were performed using the TaKaRa Multiple PCR Assay Kit Ver.2 (TaKaRa Bio Inc., Shiga, Japan) in three multiplex sets:

- Set 1: G1A, MU05, MU51
- Set 2: MU50, G10B, MU23
- Set 3: UamD2, UamB5, and sex marker SE47/48

Each multiplex PCR reaction was carried out in a total volume of 10 μL, containing 1 μL of template DNA, 0.5 μL of primer mix, 3.45 μL of nuclease-free water, 5 μL of Mix 2 (PCR buffer with dNTPs), and 0.05 μL of Mix 1 (Ex Taq DNA polymerase). Thermal cycling followed the manufacturer’s protocol: initial denaturation at 95 °C for 1 min, followed by 40 cycles of 94 °C for 30 s, 55 °C for 30 s, and 72 °C for 30 s, with a final extension at 72 °C for 10 min.

PCR products were separated by capillary electrophoresis using a SeqStudio Genetic Analyzer (Thermo Fisher Scientific, USA). Allele sizes were determined using GeneMapper™ v5.0 software with the GeneScan™ 500 LIZ size standard. Each genotype was confirmed through at least two independent PCR amplifications, and ambiguous profiles were reanalyzed.

**Mitochondrial DNA amplification and sequencing**

A short fragment of the mitochondrial control region (CR/D-loop) was amplified using bear-specific primers 161 (5′-GCGTGCCCCATGCATATAA-3′) and 162 (5′-GCTCCCGGACTAAGTGAAAT-3′) described by Lan et al. (2017). The expected amplicon length was approximately 131 bp; primer binding regions were excluded from downstream sequence alignment.

PCR reactions were conducted in a total volume of 25 μL, containing 2.5 μL of 10× Ex Taq buffer, 2 μL of dNTP mix, 1 μL of each primer (10 pmol μL⁻¹), 0.5 μL of Ex Taq polymerase (TaKaRa Bio Inc.), 17 μL of nuclease-free water, and 1 μL of template DNA. Thermal cycling conditions included an initial denaturation at 94 °C for 1 min, followed by 40 cycles of 94 °C for 30 s, 53 °C for 30 s, and 72 °C for 10 s, with a final extension at 72 °C for 5 min.

Amplification success was verified using 1.5% agarose gel electrophoresis with a non–ethidium bromide nucleic acid stain. PCR products were sequenced bidirectionally using the BigDye Terminator v3.1 Cycle Sequencing Kit (Thermo Fisher Scientific) on a SeqStudio Genetic Analyzer.

**Sequence processing and phylogenetic analysis**

Raw chromatograms were inspected for quality, ambiguous bases, and double peaks using FinchTV v1.4.0. High-quality sequences were aligned using ClustalW implemented in MEGA v12. Representative mitochondrial control-region sequences from major geographic lineages of *Ursus arctos* were retrieved from GenBank, including Tibetan brown bear, Himalayan brown bear, North American brown bear, and Hokkaido brown bear. The sloth bear (*Melursus ursinus*) was used as an outgroup.

Phylogenetic relationships were inferred using the Maximum Likelihood method under the Kimura 2-parameter model in MEGA v12. Node support was assessed using 1,000 bootstrap replicates, and branches with bootstrap support below 50% were collapsed. Initial trees were generated using Neighbor-Joining and BioNJ algorithms based on Maximum Composite Likelihood distances. All sites containing gaps or missing data were excluded from analyses.

Microsatellite genotypes were used to identify unique individuals and avoid duplicate sampling. Subspecies identification was inferred primarily from mitochondrial sequence similarity, diagnostic nucleotide substitutions, and phylogenetic clustering with reference haplotypes.
