# Supplementary material for: Confirmation of the Tibetan Brown Bear (Ursus arctos pruinosus) in Central Nepal Based on Genetic and Camera‐Trap Evidence
Source: Ecol Evol. 2026 Oct 7;16(10):e74400. doi: 10.1002/ece3.74400 (PMC13645494; doi:10.1002/ece3.74400)
Supplement: Supplementary file 2 [file ECE3-16-e74400-s002.docx]

**Table S3:** Aligned DNA Sequence

| **Mustang Samples** | **Aligned Nucleotide sequence** |
| --- | --- |
| *U.a. pruinosus*_U22CH01 | CATGTACATGTTACGCTTGGTCTTACATAAGGACTTACGTTCCGAAAGCTTGTTTTAAGTGTATGGTCTGCAAGCATGT |
| *U.a. pruinosus*_22DS01 | CATGTACATGTTACGCTTGGTCTTACATAAGGACTTACGTTCCGAAAGCTTGTTTTAAGTGTATGGTCTGCAAGCATGT |
| *U.a. pruinosus*_22DS02 | CATGTACATGTTACGCTTGGTCTTACATAAGGACTTACGTTCCGAAAGCTTGTTTTAAGTGTATGGTCTGCAAGCATGT |
| *U.a. pruinosus*_2022S2 | CATGTACATGTTACGCTTGGTCTTACATAAGGACTTACGTTCCGAAAGCTTGTTTTAAGTGTATGGTCTGCAAGCATGT |

| **Dolpa Samples** |  |
| --- | --- |
| *U.a. pruinosus*_PH76 | CATGTACATGTTACGCTTGGTCTTACATAAGGACTTACGTTCCGAAAGCTTGTTTTAAGTGTATGGTCTGCAAGCATGT |
| *U.a. pruinosus*_Sd70 | CATGTACATGTTACGCTTGGTCTTACATAAGGACTTACGTTCCGAAAGCTTGTTTTAAGTGTATGGTCTGCAAGCATGT |
| *U.a. pruinosus*_TN19 | CATGTACATGTTACGCTTGGTCTTACATAAGGACTTACGTTCCGAAAGCTTGTTTTAAGTGTATGGTCTGCAAGCATGT |
