# Supplementary material for: Confirmation of the Tibetan Brown Bear (Ursus arctos pruinosus) in Central Nepal Based on Genetic and Camera‐Trap Evidence
Source: Ecol Evol. 2026 Oct 7;16(10):e74400. doi: 10.1002/ece3.74400 (PMC13645494; doi:10.1002/ece3.74400)
Supplement: Supplementary file 3 [file ECE3-16-e74400-s003.docx]

**Table S2.** Database of two Tibetan brown bears for 8 microsatellite loci for two Tibetan brown bears in Nepal

| **Sample ID** | **Sex** | **District** | **Place/Town** | **Mt DNA** | **Bear ID** |  | **Genotype** | | | | | | | | | | | | | | | |
| --- | --- | --- | --- | --- | --- | --- | --- | --- | --- | --- | --- | --- | --- | --- | --- | --- | --- | --- | --- | --- | --- | --- |
|  |  |  |  |  |  | **Microsatellite** | **GIA_a** | **GIA_b** | **MU05_a** | **MUO5_b** | **MU52_a** | **MU52_b** | **MU50_a** | **MU50_b** | **G10B_a** | **G10B_b** | **Mu23_a** | **MU23_b** | **Uam_D2_a** | **Uam_D2_b** | **Uam B5_a** | **Uam B5_b** |
| **22CH01** | M | Mustang | Damodarkunda | Tibetan | A | Successful | 183 | 183 | 138 | 138 | 117 | 121 | 210 | 222 | 165 | 165 | 131 | 131 | 208 | 212 | 153 | 157 |
| **TN19** | M | Dolpa | Tinje | Tibetan | B | Successful | 183 | 183 | 134 | 134 | 119 | 119 | 210 | 210 | 163 | 165 | 121 | 131 | 208 | 212 | 157 | 157 |
| **Sd70Ly** | M | Dolpa | Saldang | NA | B | Successful | 183 | 183 | 134 | 134 | 119 | 119 | 210 | 210 | 163 | 165 | 121 | 131 | 208 | 212 | 157 | 157 |
| **Sd70** | M | Dolpa | Saldang | Tibetan | B | Successful | 183 | 183 | 134 | 134 | 119 | 119 | 210 | 210 | 163 | 165 | 121 | 131 | 208 | 212 | 157 | 157 |
